# Germline Stem and Progenitor Cell Aging in *C. elegans*

**DOI:** 10.3389/fcell.2021.699671

**Published:** 2021-07-08

**Authors:** Theadora Tolkin, E. Jane Albert Hubbard

**Affiliations:** Department of Cell Biology, Skirball Institute of Biomolecular Medicine, NYU Grossman School of Medicine, New York, NY, United States

**Keywords:** reproductive aging, germline flux, stem cell aging, insulin/IGF-like signal transduction pathway, Notch pathway

## Abstract

Like many animals and humans, reproduction in the nematode *C. elegans* declines with age. This decline is the cumulative result of age-related changes in several steps of germline function, many of which are highly accessible for experimental investigation in this short-lived model organism. Here we review recent work showing that a very early and major contributing step to reproductive decline is the depletion of the germline stem and progenitor cell pool. Since many cellular and molecular aspects of stem cell biology and aging are conserved across animals, understanding mechanisms of age-related decline of germline stem and progenitor cells in *C. elegans* has broad implications for aging stem cells, germline stem cells, and reproductive aging.

## Introduction

*C. elegans* is a well-established model for germline development and for aging. Several interesting related areas of study include how the *C. elegans* germ line regulates longevity (see Antebi, 2012, for review) and how reproductive cessation may relate to population success in the wild (see Hughes et al., 2007; Galimov and Gems, 2021). These will not be discussed here. Instead, we focus on the comparatively young field investigating how the *C. elegans* germ line itself ages.

In typical laboratory growth conditions, *C. elegans* hermaphrodites cease reproducing long before they die, roughly mid-way through their average 2-3 week lifespan; the average times for reproductive cessation and lifespan both depend on whether the hermaphrodites have mated (Hughes et al., 2007; Pickett et al., 2013). Among several age-related changes in the germ line, the loss of germline stem and progenitor cells is emerging as a key driver of reproductive cessation, as detailed below.

Several important aspects of *C. elegans* reproductive biology shape how reproductive aging, also referred to as reproductive senescence, is studied in this system. Therefore, prior to discussing the recent literature pertaining to the aging stem and progenitor pool, we briefly introduce the adult hermaphrodite reproductive system, Notch-mediated signaling and stem cells, and we address the effects of sperm availability in limiting reproduction. In these three introductory sections we provide only a brief overview and, at the end of each section, we direct the reader to recent reviews for additional details.

The remainder of this review covers recent work addressing the age-related decline of the germline stem and progenitor cell pool, its regulation by longevity pathways, its cellular and molecular underpinnings, and its effect on progeny production. Finally, we discuss related open questions of stem cell aging for which this model system is particularly amenable to address experimentally.

## Aspects of *C. elegans* Reproductive Biology Relevant to Reproductive Aging Studies

### Adult Hermaphrodite Reproductive System

**FIGURE 1:**
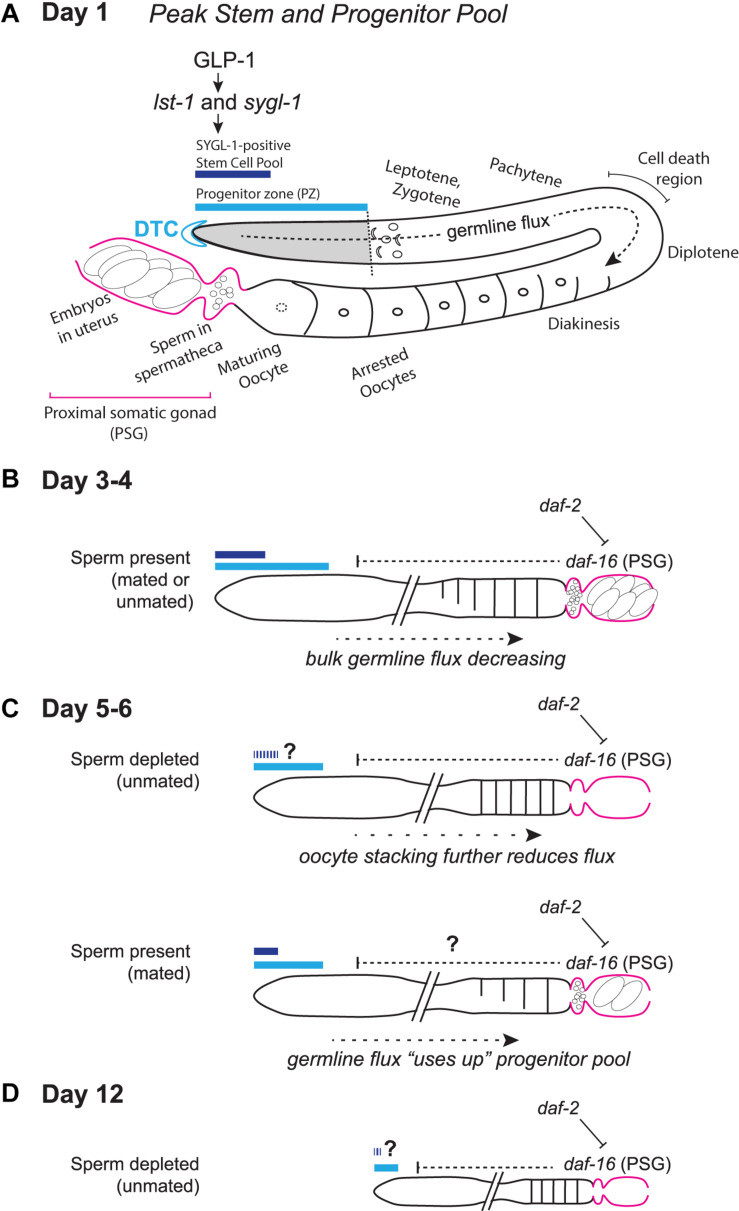
Schematic representation of the aging *C. elegans* adult hermaphrodite reproductive system. **(A)** Early adult gonad anatomy. The germline progenitor zone is located at the distal end of the gonad. As progenitors proliferate, they are displaced and move proximally (“germline flux”), progressing through oogenic prophase of meiosis I until they become arrested in diakinesis at the proximal end of the oviduct, awaiting maturation-inducing signals from sperm in the spermatheca. **(B)** Simplified schematic of a days 3–4 adult gonad, depicting distal stem/progenitor pool and proximal arrested oocytes, sperm in the spermatheca and embryos in the uterus. **(C)** Schematics representing aged (days 5–6) sperm-depleted and sperm-replete conditions. **(D)** Schematic representing a post-reproductive day 12 gonad. Regardless of sperm availability, reproduction has ceased, and the progenitor zone is severely reduced. Diagrams in **(A–D)** represent wild-type worms. Additional notes: The progenitor zone of aged hermaphrodites contains fewer cells than at adult day 1 peak; in mated hermaphrodites (as yet untested in the other conditions) SYGL-1- and LST-1- positive pools also decline. Sperm-depleted hermaphrodites accumulate unfertilized oocytes in the oviduct, whereas sperm-replete hermaphrodites do not. The PZ mitotic cell cycle also slows, as does the rate of meiotic entry and the rate of progression through prophase of meiosis I. These age-related changes in bulk germline flux contribute to the reduction in progeny production with age. The numbers of oocytes are schematized here to emphasize flux vs. stacking; Kocsisova et al. (2019) report an average of ∼14 oocytes are in diplotene and diakinesis at days 1 and 3 and an average of ∼9 at day 5 in mated hermaphrodites. See text for details and additional references.

Most *C. elegans* aging studies have been conducted in the hermaphrodite, a morphological “female” that produces sperm for a short time at the end of larval life before switching solely to oocyte production. The tube-shaped adult hermaphrodite gonad is closed at the distal end, and opens to the uterus at the proximal end (Figure 1A). Hermaphrodites bear two gonad “arms” (one anterior and one posterior) that open to a single uterus. A pool of proliferating germ cells resides at each distal (closed) end, in a region dubbed the progenitor zone (PZ). The PZ pool of cells accumulates during larval stages and reaches a maximum number of cells in early adulthood. The PZ contains germline stem cells, their proliferating progeny, and cells in meiotic S phase. Cells enter prophase of meiosis I as they leave this zone.

Each germ cell nucleus is surrounded by its own plasma membrane, including nuclei in the distal zone, but germ cells retain an opening to a core (the rachis) into which contents flow proximally to supply growing oocytes. As many as 85% of adult germ cells that enter prophase of meiosis I have been estimated to later undergo apoptosis in late pachytene. This cell death is thought to constitute a nurse cell function providing material for growing oocytes via the core.

Adult germ cells that do not undergo programmed cell death continue to mature as oocytes, arresting in diakinesis while awaiting their “turn” to reach the proximal-most position in the oviduct (Figure 1). In response to hormone signals from sperm residing in the spermatheca (located just proximal to the oviduct), oocytes escape arrest, undergo meiotic maturation, and are ovulated into the spermatheca where they are fertilized. Each zygote then moves into the uterus and begins embryonic development. For details on germline development and oocyte maturation, the reader is directed to recent reviews and additional references therein (Das and Arur, 2017; Hillers et al., 2017; Huelgas-Morales and Greenstein, 2018; Hubbard and Schedl, 2019).

### Notch Signaling and Germline Stem Cells

Maintenance of *C. elegans* germline stem cell identity depends on canonical Notch signaling. Each distal end of the hermaphrodite gonad is capped by single somatic cell called the distal tip cell (DTC; Figure 1A). The DTC produces Delta-like ligands that activate a Notch family receptor, GLP-1, that is present on the surface of germ cells in the PZ. GLP-1 activity in the distal germ cells activates the transcription of two genes, *sygl-1* and *lst-1*, the products of which act largely redundantly to prevent differentiation. SYGL-1-positive cells therefore can be considered stem cells, as distinct from SYGL-1-negative cells in the PZ that cycle through a last mitosis before entering meiotic S phase. Since SYGL-1 and LST-1 were relatively recently discovered and characterized as the sole early response to GLP-1 signaling (Kershner et al., 2014; Shin et al., 2017; Haupt et al., 2019; Chen et al., 2020), and since techniques (such as those for single-copy insertions necessary for transgene expression in the germ line and for CRISPR/Cas9-mediated tagging) have only relatively recently gained widespread use in the field, only one aging study (Kocsisova et al., 2019) has thus far distinguished stem vs. non-stem pools within the aging PZ. For details on GLP-1 Notch signaling in the germ line and additional details of the *C. elegans* stem cell system, the reader is directed to recent reviews and additional references therein (Greenwald and Kovall, 2013; Kershner et al., 2013; Hubbard and Schedl, 2019).

### Sperm Limitation and Oocyte Maturation

Two additional features of *C. elegans* reproductive biology are that sperm are limiting for self-progeny production, and that sperm are required to stimulate oocyte maturation (see Huelgas-Morales and Greenstein, 2018). Self-sperm that are generated in the last larval stage of hermaphrodite development fertilize oocytes one by one as oocytes are ovulated into the spermatheca. Sperm are thereby depleted over time, and unmated hermaphrodites will produce effectively the same number of self-progeny as they have self-sperm. Unmated hermaphrodites cease reproduction after ∼4–5 days having produced ∼300 self-progeny. Thereafter, oocytes arrested in diakinesis stack up in the oviduct. These oocytes are occasionally ovulated into the uterus and can be expelled from the animal through the vulva. Unfertilized oocytes that remain in the uterus can also become endomitotic. Thus, the cessation of reproduction in unmated hermaphrodites in the lab is mainly driven by sperm depletion, not reproductive or germ cell aging *per se*.

The limit to self-progeny production can be circumvented if a hermaphrodite mates with a male. In this scenario, abundant sperm are present and arrested oocytes do not stack up in the oviduct. Rather, oocytes are continuously matured and ovulated in response to sperm signals. The two different scenarios—one in which oocytes remain arrested in the absence of sperm, and one in which oocytes are continuously maturing and ovulating—represent different scenarios for worm biology and for reproductive aging as defined by progeny production (Pickett et al., 2013).

## The Germline Stem and Progenitor Pools Decline with Age

Given the complex biology of sperm limitation and oocyte maturation, a major finding that has emerged from recent studies is that the number of germ cells in the progenitor zone (PZ) declines with age. This occurs in both unmated and mated hermaphrodites despite the profound gene expression changes that accompany sperm depletion (Angeles-Albores et al., 2017). In both scenarios, this PZ decline begins early in adulthood, during peak reproduction, and before somatic aging becomes apparent (Garigan et al., 2002; Killian and Hubbard, 2005; Hughes et al., 2007; Luo et al., 2010; Narbonne et al., 2015; Qin and Hubbard, 2015; Kocsisova et al., 2019). Discrepancies regarding exact PZ numbers (see a summary in Materials and Methods of Kocsisova et al., 2019), likely reflect different laboratory conditions and strains, as well as different analysis and imaging techniques. For example, dissected gonads from very old worms are fragile, possibly unintentionally biasing results from such preparations to gonads from less fragile worms in an aging population. Nevertheless, the studies discussed below confirm that PZ decline is a hallmark feature of aging, and that depletion of the stem/progenitor pool is a major driver of the age-related decline in reproductive output.

### Insulin/IGF-Like Receptor Signaling (IIS) and Decline of the Germline Progenitor Pool With Age

Not long after the groundbreaking discovery that *daf-2*, which encodes the sole insulin/IGF-like receptor in *C. elegans*, promotes aging, a role was postulated for this gene and its signaling pathway (hereafter abbreviated as IIS) in reproductive aging. Reduction-of-function (*rf)* mutations in *daf-2* extend lifespan in a manner dependent upon the transcription factor DAF-16 FOXO (see Kenyon, 2010 for review). That *daf-2* influences germ cell aging was suggested by a qualitative examination and rating of germ cell morphology in *daf-2* and *daf-16* mutants (Garigan et al., 2002). A quantitative measure associated with germline aging, a decline in the number of PZ cells in wild-type adult hermaphrodites over time, was subsequently documented in fixed whole-mount preparations (Killian and Hubbard, 2005), and this observation has been confirmed by studies in several different labs (Luo et al., 2010; Narbonne et al., 2015; Qin and Hubbard, 2015; Kocsisova et al., 2019). Reducing IIS slows the age-related decline in the number of cells in the PZ, and this slower depletion of the PZ pool in *daf-2* mutants is dependent on *daf-16* (Luo et al., 2010; Qin and Hubbard, 2015).

One hypothesis that was considered for why germline progenitor pool depletion might be delayed in the *daf-2* long-lived mutant is that whatever mechanisms extend lifespan might slow germline aging as measured by PZ depletion. However, this explanation proved unlikely since the tissues that require wild-type *daf-16* activity to promote longevity downstream of *daf-2* are not the same as those that require wild-type *daf-16* activity to affect depletion of the PZ. That is, tissue-specific expression of *daf-16*(+) in the intestine of the worm partially reverses the effects of *daf-16* loss on lifespan of the *daf-16; daf-2* double mutant, making it more similar to *daf-2* alone (Libina et al., 2003). However, animals carrying the same transgene showed a PZ pool similar to the *daf-16; daf-2* double mutant rather than to the *daf-2* mutant alone at day 12 of adulthood. Nor does transgenic expression of *daf-16*(+) in muscle or neurons influence age-related PZ loss in the *daf-16; daf-2* double mutant (Qin and Hubbard, 2015).

Instead, unexpectedly, *daf-16*(+) is required in certain cells of the proximal somatic gonad (PSG), a source of DAF-16 not previously linked to longevity nor to PZ regulation. Specifically, expression of *daf-16*(+) in a subset of (*fos-1a-*positive) PSG cells of the spermatheca and uterine lineage completely reversed the effect of the *daf-16* mutant such that the double mutant *daf-16; daf-2* expressing *daf-16*(+) in PSG cells maintained a larger PZ pool with age, similar to the *daf-2* mutant alone. Consistent with this finding, tissue-specific RNAi depletion of *daf-16* in the same PSG cells mimicked loss of *daf-16*, while depletion of *daf-16* in the germ line did not (Qin and Hubbard, 2015). This last result was also somewhat surprising since the *daf-2* signaling pathway is required germline-autonomously to promote accumulation of the PZ pool during larval stages (Michaelson et al., 2010). One possible explanation for these results was that these PSG cells represented an undiscovered source of *daf-16*(+) activity in promoting longevity. However, this possibility was ruled out since lifespan is unaffected in *daf-16; daf-2* double mutant worms bearing the PSG-expressed *daf-16*(+) transgene. Thus, age-related germline progenitor pool decline and organismal lifespan can be modulated by the same IIS signaling pathway, but from anatomically distinct activities of *daf-16* in the worm (Qin and Hubbard, 2015).

### The Progenitor Zone Is “Used Up” by Germline Flux

The unexpected role for DAF-16 in PSG cells opened a number of questions regarding the mechanism by which the PZ is depleted with age. In short, the results support a model in which the progenitor pool is “used up” over time. An analysis of mutants that interfere with sperm production, ovulation, or fertilization suggested that the rate at which germ cells leave the system by oocyte ovulation influences the rate of PZ loss. That is, preventing ovulation slows the PZ pool decline. This effect is seen in sperm-less mutants in which oocytes stack up in the oviduct, and the effect can be partially reversed by reintroducing sperm. Importantly, the maintenance of the PZ over time that occurs in the absence of sperm is partially dependent on *daf-16* in the PSGs (Qin and Hubbard, 2015). In addition, local germline-autonomous feedback slows cell cycle progression in the PZ pool in response to accumulating oocytes, in a manner dependent on *daf-18*, which encodes the *C. elegan*s ortholog of human tumor suppressor PTEN (Narbonne et al., 2015; see also below).

### Time-Course and Cellular Mechanisms of Progenitor Pool Decline

The decline in the number of PZ cells begins early, by day 3 of adulthood, coincident with peak reproductive output and before various measures of somatic decline are evident (Killian and Hubbard, 2005; Pickett et al., 2013; Kocsisova et al., 2019). While estimates vary in terms of exact cell number, in both unmated and mated hermaphrodites, the PZ declines from ∼200 to 250 cells at day 1 of adulthood to ∼100 to 150 cells by day 6. PZ cell numbers continue to decline during adulthood, such that an average of ∼50 PZ cells remain on adult day 12 in unmated hermaphrodites (Qin and Hubbard, 2015). The time-course of progeny production, in both unmated and mated hermaphrodites shows an early peak and then steep decline: in either case, an average of ∼150 progeny are produced on adult day 2, while very few progeny are produced after day 5 in unmated and after day 8 in mated hermaphrodites (Pickett et al., 2013; Kocsisova et al., 2019).

The number of cells in the PZ at any given time is a function of several factors, the interdependence of which is not fully resolved. These factors include the starting number of stem cells and non-stem cells in early adulthood, the rate of proliferation of each of these pools within the PZ, the rate of entry of cells into the meiotic pathway by commitment to meiotic S phase, the duration of meiotic S phase, and entry into prophase of meiosis I. Each of these factors may or may not change in concert over time. The rate of particular phases of mitotic cell cycle progress is, in particular, difficult to measure, requiring arduous pulse-chase time-course analysis (e.g., Fox et al., 2011; Kocsisova et al., 2019; see below). Mitotic index—the number of cells in metaphase divided by the number of proliferation-competent cells in a pool—is a proxy for the rate of cell cycle progression since, in theory, a higher mitotic index in a population of cells would reflect a faster average rate of cell cycle progression. However, compensatory alterations of other cell cycle phases or cell populations that are not uniform can potentially confound the interpretation of mitotic index alone. In addition, the length of time that *C. elegans* germ cells express a common mitotic marker (phospho-histone H3) is itself subject to nutritional state (Gerhold et al., 2015). Another mitotic index measure that gives a lower but perhaps more reliable index is the proportion of cells in metaphase or anaphase of mitosis. These concerns notwithstanding, by either measure, cells in the PZ of self-fertile hermaphrodites display a reduction in mitotic index as sperm become depleted (Narbonne et al., 2015; Qin and Hubbard, 2015).

While GLP-1 is not thought to regulate cell cycle, stem cell identity and cell cycle dynamics are not completely independent. As germ cells are displaced away from the distal end, they lose contact with the DTC and lose GLP-1 activity. Since germ cells spend the majority of time in S and G2, after these cells lose GLP-1 activity they must usually cycle through M phase before entering meiotic S (Fox and Schedl, 2015). As a result, a prolonged G2 of an aged germ cell cycle may appear to inhibit meiotic entry. An extreme example is seen in germ cells such as those in adult reproductive diapause (ARD) that are essentially arrested (Angelo and Van Gilst, 2009; Seidel and Kimble, 2011). These cells do not enter meiotic prophase even in the absence of GLP-1 Notch signaling (Seidel and Kimble, 2015), likely because they cannot progress to meiotic S.

What forestalls the age-related decline in the PZ pool when *daf-2* is reduced or when germline flux is decreased upon sperm depletion? One factor is likely to be slower cell cycle progression. Introducing sperm to sperm-depleted hermaphrodites can accelerate the decline of PZ cells (Qin and Hubbard, 2015) and elevate mitotic index (Narbonne et al., 2015). Together, these results suggest that the rate of cell proliferation is an important factor dictating the number of PZ cells at a given time. Reduced *daf-2* and slowed oocyte flux both correlate with lower mitotic index in early adulthood (Narbonne et al., 2015). Day 1 mitotic index is lower in conditional *daf-2* mutants at the restrictive temperature regardless of mating status, while sperm-less mutants display reduced mitotic index that is partially reversed by mating. In both cases, loss of DAF-18 PTEN, a phosphatase that can oppose the PI3 kinase activity downstream of DAF-2, restores mitotic index. Based on manipulations that selectively allow or block flux in one gonad arm or the other within the same hermaphrodite, Narbonne et al. (2015) postulate that accumulated oocytes themselves locally antagonize DAF-2-mediated signaling via DAF-18 PTEN to slow the cell cycle in the same gonad arm. Interpretation of some of these experiments is complicated, however, especially those that use tumor-forming mutants in which the propensity to form tumors and the rate of proliferation within tumors are not necessarily correlated (see Hubbard and Schedl, 2019, for a discussion of tumorous mutants). Based on genetic analysis, DAF-18 is proposed to act upstream of AMPK and MAP kinase signaling (Narbonne et al., 2017). In this model, the logjam of arrested, un-ovulated oocytes in the proximal oviduct would signal either directly or indirectly to the distal germ line to slow the cell cycle and/or modulate progression into meiosis I prophase (see also Discussion).

### Decline of the Progenitor Pool Is a Main Contributor to Reproductive Decline With Age

To understand how changes to the progenitor pool ultimately relate to progeny production, it is necessary to analyze hermaphrodite reproductive aging in sperm-replete conditions. This is because sperm depletion limits progeny production and introduces confounding effects due to the build-up of arrested oocytes upon sperm depletion. Therefore, Kocsisova et al. (2019) examined reproductive aging in mated worms. This comprehensive study demonstrated that PZ depletion does indeed occur in sperm-replete conditions, that it includes a depletion of the SYGL-1-positive stem cell pool, that it begins early relative to somatic aging, and that it is a primary contributor to reduced progeny production with age. Although oocyte quality is controlled by multiple mechanisms (e.g., Nadarajan et al., 2009; Luo et al., 2010; Templeman et al., 2018; see also below; Bhargava et al., 2020; Templeman et al., 2020), oocyte quantity, and hence the number of progeny, is tightly correlated with output from the PZ pool (Agarwal et al., 2018; Kocsisova et al., 2019).

Overall, a 14-fold reduction in progeny production occurs in mated worms by adult day 7 compared to adult day 1, and the reduction in progeny production follows PZ depletion with a delay of ∼2.5 days, the time required for cells to go from PZ to oocyte (Kocsisova et al., 2019). The observation that the PZ in worms with reduced *glp-1* activity declines faster suggested that loss of the GLP-1-responding stem cell pool contributes to PZ decline (as opposed to, for example, only a loss of non-stem progenitors) (Qin and Hubbard, 2015). A *bona fide* stem cell decline was demonstrated by Kocsisova et al. (2019) using CRISPR/Cas9-generated tagged SYGL-1 and LST-1 proteins. A reduced SYGL-1-positive stem cell pool was seen as a shortening of the proximal extent of SYGL-1 protein detection, and that the decline was due to reduced GLP-1 signaling was indicated by a decreased proximal extent of LST-1 protein. Together, stem cell decline is ∼2-fold by adult day 5 (Kocsisova et al., 2019).

Cell cycle analysis in mated hermaphrodites, measuring M and S phase index as well as G2 duration and total non-S duration, indicated that between days 1 and 3 of adulthood, the duration of the G2 is extended ∼2 fold (2.5–4.9 h), as is the entire non-S portion of the cell cycle (G2 + M + G1; 3.4–7.4 h). Interestingly, neither the M-phase index nor S-phase index change over this timeframe in mated worms. One possible explanation is that pro-metaphase may be lengthened as well, as these studies used anti-phospho-histone H3 to measure mitotic index. Taking all these data into account, the average speed of the cell cycle is reduced ∼2-fold by day 3 (Kocsisova et al., 2019).

In addition to PZ cells lengthening certain cell cycle stages in aging mated worms, the rate at which they enter meiosis is also reduced, as indicated by pulse-chase analysis of EdU-labeled germ cells (Kocsisova et al., 2019). In general, how the rate of meiotic entry is controlled is unknown. However, regulation could occur at several different levels since multiple pathways control the decision to enter the meiotic pathway upon loss of GLP-1 signaling (see Hubbard and Schedl, 2019). Taken together, the change in the total output of the PZ, including stem cell depletion, reduced cell cycle, and reduced meiotic entry, is estimated as ∼4.5-fold by day 5 and ∼11-fold by day 7, relative to peak (Kocsisova et al., 2019).

In addition to a slower rate of entry into prophase of meiosis I, progress through meiotic prophase also slows with age. Aging extends time spent in leptotene/zygotene as well as pachytene (Jaramillo-Lambert et al., 2007; Kocsisova et al., 2019). However, the same proportion of cells were estimated to undergo physiological apoptosis at day 1 as at day 5. Thus, an additional delay in progression through the various stages of prophase of meiosis I, rather than an increase in the proportion of cells that die, likely contributes to the mid-life reduction in progeny production (Kocsisova et al., 2019).

Among a number of germline aging phenotypes, the age-related reduction of the PZ pool occurs in essentially all individuals within the aging population, both mated and unmated hermaphrodites (Luo et al., 2010; Narbonne et al., 2015; Qin and Hubbard, 2015; Kocsisova et al., 2019). In addition, this age-related phenotype occurs similarly in both gonad arms within individuals, arguing for a systemic rather than a local effect (Kocsisova et al., 2019). In contrast, two additional sporadic low-frequency aging phenotypes are observed in mated hermaphrodites: a shift of the DTC nucleus position and endomitotic oocytes in the oviduct (as distinct from endomitotic oocytes in the uterus which accumulate in hermaphrodites that have exhausted their sperm supply). Longitudinal studies of hermaphrodites displaying these two infrequently-observed defects indicate that the two are not correlated and, while a shifted DTC nucleus does not strongly correlate with reproductive decline, the presence of endomitotic oocytes in the oviduct of mated worms negatively impacts reproduction. In addition, the DTC phenotype was often restricted to one gonad arm and not the other within individuals, while the presence of endomitotic oocytes in one gonad arm in an individual was correlated with an increased probability of observing an endomitotic oocyte in the other gonad arm of the same individual, suggesting a local mechanism for the former and a possible systemic effect for the latter (Kocsisova et al., 2019).

## Discussion and Open Questions

Many open questions remain regarding germline stem and progenitor cell aging, and many of these open questions are experimentally accessible in *C. elegans* (Corsi et al., 2015). The system is both fast-reproducing and fast-aging. It is amenable to unbiased genetic screening and single-cell omics. Moreover, cell and tissue autonomy can be rigorously established, developmental vs. aging effects can be separated using conditional alleles or other conditional manipulations, and longitudinal studies of individual worms using live markers are feasible since the worms are transparent. Most important, many cellular and molecular aspects of aging and stem cells are conserved between *C. elegans* and mammals (see Hubbard and Schedl, 2019). Therefore, studying the aging germ line in this system is likely to further our understanding of stem cell aging in general and reproductive aging in particular. Below we discuss some of these open questions and associated hypotheses.

### How Is the Germ Cell Cycle Regulated During Aging?

Like germ cells of other organisms (Hsu et al., 2008; Ables and Drummond-Barbosa, 2013; Kao et al., 2015) and like mammalian embryonic stem cells (White and Dalton, 2005), *C. elegans* germ cells display unusual cell cycle control, including a very short to no G1 (Fox et al., 2011; Lara-Gonzalez et al., 2019). Although CDK-2/CYE-1 Cyclin E is required for cell cycle progression, in contrast to somatic cells where CDK2/Cyclin E is typically limited to late G1 and early S phase, the complex is present in germ cells at high levels throughout the mitotic germ cell cycle, where it also has a role in promoting the proliferative fate (Fox et al., 2011). Interestingly, a similar situation exists in *Drosophila* for Cyclin E (Hsu et al., 2008; Ables and Drummond-Barbosa, 2013), where S phase delay occurs in aging germline stem cells (Kao et al., 2015). In *C. elegans* germ cells, GSK-3 glycogen synthase kinase is required to maintain high levels of *cdk-2* transcription throughout the cell cycle to promote a high rate of cell cycle progression (Furuta et al., 2018). The unusual nature of the germline cell cycle necessitates careful characterization of cell cycle markers. For example, a well-characterized marker for S-phase, PCNA or PCN-1 in *C. elegans*, can accumulate in all PZ nuclei throughout the cell cycle, when only about half are in active S-phase as measured by EdU labeling (Kocsisova et al., 2018). A deeper understanding of the mechanism of stem cell cycle regulation in the germ line, together with well-characterized markers for cell cycle stages, will facilitate experiments needed for a more thorough understanding of how the germ cell cycle changes with advancing age.

The negligible G1 necessitates germ cell cycle regulation in other phases of the cycle. In germ cells, G2 arrest occurs in nutrient-poor conditions in several developmental scenarios, including the first larval stage, dauer and ARD (e.g., Fukuyama et al., 2006; Seidel and Kimble, 2015; Tenen and Greenwald, 2019), and the G2 is extended in older mated hermaphrodites (Kocsisova et al., 2019). One question is whether IIS, which promotes PZ mitotic index in larval and adult germ cells (Michaelson et al., 2010; Narbonne et al., 2015; Roy et al., 2016), is involved in the extension of G2 with age. If so, does it share mechanisms of IIS-mediated G2 regulation that occur in *Drosophila* neural stem cells (Otsuki and Brand, 2018)? Which of the many *C. elegans* insulin/IGF-like ligands are responsible, and how are they regulated by age? If not IIS, what mechanisms cause the extension of G2 with age?

Even though the average rate of cell cycle progression, which reduces the number of cells born over time, and the average rate of meiotic entry are both slowed with age, the number of cells in the PZ declines with age in either sperm-depleted or sperm-replete conditions. This suggests that the two rates of change are unequal; that is, either they start or progress differently over time. The rate of change in the cell cycle within the stem cell vs. non-stem progenitor pools may also differ. Nevertheless, stem cell depletion is likely a major factor in overall PZ decline since fewer stem cells results in fewer progenitors that can enter meiosis. The stem vs. progenitor state and cell cycle status of cells that remain in the PZ in oldest worms is also unresolved.

### How Is Stem Cell Fate and Function Altered With Age?

The age-related decline in the pool of germ line stem cells is defined by the reduced number of the GLP-1 Notch responsive (SYGL-1-positive) cells in the distal germ line. One possibility is that this conserved Notch signaling pathway is affected by age-related cellular and tissue-level changes. Since Notch activity regulates stem cell fate in several different mammalian organ contexts, worm studies may inform our understanding of stem cell aging in higher organisms as well. Even in cases where other (non-Notch) signaling pathways control stem cell fate, features of stem cell signaling in the context of aging tissues may well be conserved.

Notch activation in the *C. elegans* germ line occurs via membrane-bound ligand expression in the somatic niche cell and the GLP-1 receptor on the surface of germ cells. Why does the number of SYGL-1-expressing stem cells decrease as animals age? Are the levels of Notch signaling in the stem cells reduced? If so, at what step of the signaling pathway? Is the post-transcriptional regulation of Notch pathway components or effectors different in old worms than in young ones? How do changes in Notch signaling interface with the changes in the germ cell cycle? To what extent is the reduction in Notch signaling due to general age-related changes to cell membranes or signaling centers? Is the efficacy of signal reception or transmission affected by age-related alterations in receptor membrane localization or receptor processing? Is the reduction in Notch signaling due to aging in the soma or in the germ line? Do age-related changes in nuclear transport or germ cell chromatin interface with transcriptional activation downstream of receptor signaling? Do changes to Notch pathway signaling reflect interactions with other age-related signals that impinge on the germ line? If so, how?

Equally interesting will be an understanding of how specific aspects of cell biological aging, such as changes to the basement membrane, to mitochondrial function, or to proteostasis, contribute to the decline in stem cell fate and function. It is possible, for example, that some aspects of cellular aging impact cell cycle while others impact cell fate decisions via effects on specific signal transduction pathways.

### How Does IIS Regulate the PZ? Do Other Aging Pathways Prevent PZ Decline?

With respect to IIS, organismal lifespan and the germline progenitor pool are regulated in anatomically distinct ways. How does *daf-16* expression in a subset of proximal somatic gonadal cells regulate the rate of decline of the PZ pool? One hypothesis is that a *daf-16*-dependent secreted factor signals directly to the germ line. Alternatively, PSG-to-germline communication could occur through intermediate tissues. The relevant targets of *daf-16* in the PSG will also be important to identify. To what extent does this pathway act at the level of germline stem cell identity, cell cycle, or both? It will also be of interest to determine how other age-defying conditions and genetic manipulations influence PZ aging, and, if so, whether they act by mechanisms similar to or distinct from the *daf-2* pathway.

Interfering with germ cell flux also delays the decline of PZ cell numbers with age. This effect appears to be linked to ovulation of oocytes and is partially dependent on *daf-16* in the proximal somatic gonad (Qin and Hubbard, 2015) as well as *daf-18*, AMPK and MAP kinase (Narbonne et al., 2015, 2017). Whether these pathways act directly or indirectly on the PZ requires further investigation.

### How Is PZ Decline Related to Oocyte Quality?

By about day 5 of adulthood, oocytes show overt signs of deterioration (Garigan et al., 2002; Luo et al., 2010). They become morphologically disordered, and the progeny they give rise to show a higher incidence of chromosome non-disjunction and embryonic lethality, even if the old hermaphrodite is provided with sperm from a young male (Killian and Hubbard, 2001; Luo et al., 2010; Templeman et al., 2018; Imakubo et al., 2021). Though never fully avoided, defects in late life reproductive quality are delayed in long-lived mutants. However, the rate of egg-laying is also reduced throughout the life-span of long-lived mutants (Pickett et al., 2013), so one possibility is that oocyte deterioration will always occur after a certain number of oocytes have been produced, rather than at a particular biological age of the worm. Given the long journey a germ cell makes from stem cell to oocyte, it is also likely that age-dependent oocyte defects result from age-related changes in multiple factors and processes distributed throughout the course of ongoing germ cell maturation, starting from the stem cells themselves.

An interesting and potentially relevant observation is that reducing GLP-1 signaling in the distal germ line regulates the size of oocytes: reducing *glp-1* activity results in large oocytes, while elevating *glp-1* activity results in small oocytes (Nadarajan et al., 2009). This effect of *glp-1* on oocyte size is dependent on DTC-mediated GLP-1 activation, gap junctions, and cell death. It is mediated by alterations in the rate of actomyosin streaming to oocytes as well as the timing of oocyte cellularization. These observations demonstrate that the effects of GLP-1 signaling activity in the PZ, in addition to regulation of stem cell numbers, may have downstream effects on oocytes relevant to oocyte quality in aging.

A related question is: does PZ decline affect subsequent progression through prophase of meiosis I? Or are these independently regulated processes? Although age does not appear to affect physiological cell death during prophase, it does influence the rate of progression through prophase (Kocsisova et al., 2019). Progression through several distinct points of prophase of meiosis I is regulated by DAF-2 and RAS/MPK signaling (see Das and Arur, 2017 for a review). That oocytes arrested in late prophase may feed back on the PZ via DAF-18 PTEN, via AMPK and MAPK has been proposed (Narbonne et al., 2015, 2017), however, it is at present difficult to untangle the causal links between these pathways vis-à-vis the PZ and meiotic prophase progression, since *daf-2*, *daf-18*, and *mpk-1* all act at multiple steps in both processes. Additional questions concern how reducing the progenitor pool or slowing meiotic prophase affects the quality and quantity of material that eventually accumulates in oocytes.

### How Is PZ Decline and Reproductive Aging Affected by the Worm’s Environment?

*C. elegans* is a powerful model for understanding how an organism’s environment, physiology and genetics together dictate phenotype. *C. elegans* research continues to advance genetic understanding of virtually every aspect of development and cell biology, largely under highly controlled laboratory conditions using a mono-species abundant food source. However, the worm’s environment—microbial (both quality and quantity), pheromone, and temperature—impacts many aspects of development. A well-understood example is the regulation of dauer, an alternate pre-reproductive larval stage that is resistant to poor environmental conditions (see Fielenbach and Antebi, 2008 for a review). Interestingly, molecular pathways that regulate dauer such as IIS, TGFβ, and nuclear hormone signaling pathways, also affect both aging and the germ line, even when worms have not experienced dauer (e.g., Michaelson et al., 2010; Dalfó et al., 2012; Thondamal et al., 2015; Pekar et al., 2017), and IIS regulates meiotic progression in response to nutrient availably (Lopez et al., 2013).

Aging and reproduction are particularly sensitive to environmental manipulation in *C. elegans*. Under extreme conditions of starvation in late larval stages, reproductive adults can arrest germline stem cell divisions and slow the rate of oocyte production from the usual ∼3 oocytes ovulated per hour per gonad arm in peak times to fewer than one every 8 h (Angelo and Van Gilst, 2009; Seidel and Kimble, 2011). Remarkably, worms can remain in this condition far longer than their normal lifespan and rebound to become reproductively competent adults with a functional, albeit reduced, PZ (Roy et al., 2016). Responses to other stressors such as oxidative and heat stress are also altered with age, but it is unclear how they affect germline aging.

Slowing the germline progenitor cell cycle during environmentally unfavorable conditions or after sperm depletion may be an evolutionary strategy to allow sperm-depleted hermaphrodites to temporarily retain germline progenitors despite advancing age, such that PZ cell proliferation, entry into and through meiotic prophase, and oocyte production can resume quickly upon locating new food sources and/or males with which to mate.

## Concluding Remarks

The quirks of *C. elegans* life history and reproductive strategies have been sculpted by evolution to ensure its success in the wild. Nevertheless, together with its advantages as a laboratory model organism, these quirks offer rich opportunities for experimental analysis of reproductive and stem cell aging. Given the conservation of cellular and molecular processes from worms to mammals, and given the history of what model organism research has contributed to fundamental biology relevant to human health, studies on germline stem cell aging should offer broad implications beyond the worm.

## Author Contributions

Both authors contributed to the article and approved the submitted version.

## Conflict of Interest

The authors declare that the research was conducted in the absence of any commercial or financial relationships that could be construed as a potential conflict of interest.
